# Long-Term Stability and Optoelectronic Performance Enhancement of InAsP Nanowires with an Ultrathin InP Passivation Layer

**DOI:** 10.1021/acs.nanolett.2c00805

**Published:** 2022-04-14

**Authors:** LuLu Chen, Stephanie O. Adeyemo, H. Aruni Fonseka, Huiyun Liu, Srabani Kar, Hui Yang, Anton Velichko, David J. Mowbray, Zhiyuan Cheng, Ana M. Sanchez, Hannah J Joyce, Yunyan Zhang

**Affiliations:** † School of Micro-Nano Electronics, Zhejiang University, Hangzhou, Zhejiang 311200, China; ‡ Electrical Engineering Division, Department of Engineering, University of Cambridge, 9 JJ Thomson Avenue, Cambridge CB3 0FA, United Kingdom; # Department of Physics, University of Warwick, Coventry CV4 7AL, United Kingdom; § Department of Electronic and Electrical Engineering, University College London, London WC1E 7JE, United Kingdom; ∥ Institute for Materials Discovery, University College London, Roberts Building, Malet Place, London, WC1E 7JE, United Kingdom; ¶ Department of Physics and Astronomy and the Photon Science Institute, University of Sheffield, Sheffield S3 7RH, United Kingdom

**Keywords:** thin nanowire, surface passivation, ultrathin InP, long-term stability, photonic properties

## Abstract

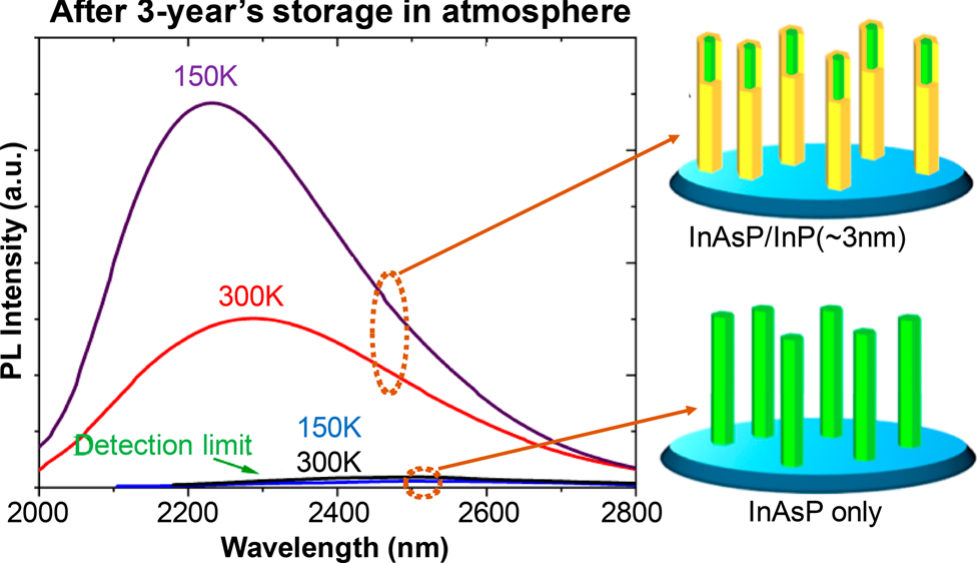

The influence of nanowire (NW) surface states increases rapidly with the reduction of diameter and hence severely degrades the optoelectronic performance of narrow-diameter NWs. Surface passivation is therefore critical, but it is challenging to achieve long-term effective passivation without significantly affecting other qualities. Here, we demonstrate that an ultrathin InP passivation layer of 2–3 nm can effectively solve these challenges. For InAsP nanowires with small diameters of 30–40 nm, the ultrathin passivation layer reduces the surface recombination velocity by at least 70% and increases the charge carrier lifetime by a factor of 3. These improvements are maintained even after storing the samples in ambient atmosphere for over 3 years. This passivation also greatly improves the performance thermal tolerance of these thin NWs and extends their operating temperature from <150 K to room temperature. This study provides a new route toward high-performance room-temperature narrow-diameter NW devices with long-term stability.

Nanowires (NWs) feature a quasi-one-dimensional morphology and have many potential novel applications, including light emitters, photovoltaics, and high speed electronics.^1−5^ They have significant advantages over thin films/bulk III–V materials, as they can be readily integrated on silicon (Si) substrates with high quality, because their small contact area with the substrate confines the strain relaxation-formed dislocations to the vicinity of the NW/substrate interface. This means not only great flexibility in device design and potential for low-cost fabrication, but also seamless integration with the silicon industrial platform, solving the III–V/Si integration challenge that has been pursued for more than 40 years.^6,7^ Among different NWs, InAsP NWs have a narrow and direct band gap that can extend nanophotonic applications from near-infrared (∼922 nm) to the mid-infrared spectral region (3.5 μm), and is an ideal material system for broadband photodetectors for applications in optical communications, surveillance, thermophotovoltaics, and thermal imaging. It is also a good candidate to build high-efficiency light sources on Si, such as 1.3 and 1.5 μm lasers and single photon sources that will have important applications in Si photonics, quantum computing, and quantum communication.

NWs have a large surface-to-volume ratio which can provide a greatly enlarged effective working area. For example, when used for photovoltaics, they can provide a junction area that is much larger than the cross section of the NWs, which is beneficial for efficient carrier (electrons and holes) separation and collection.^8^ Unfortunately, the large surface-to-volume ratio of NWs also exacerbates the influence of surface states that can cause severe Fermi-level pinning, depletion of charge carriers, increased charge carrier scattering, and high nonradiative surface recombination rates, leading to slow device response and low efficiency.^9,10^ The influence of the surface states increases rapidly with the surface-to-volume ratio when the NW diameter reduces. The surface states can even deplete the whole volume of thin NWs, rendering them unusable for devices.^11,12^ Furthermore, III–V materials, especially high-InAs-content ones with a small band gap, have small charge carrier effective masses and hence high charge carrier mobilities, so carriers easily reach the NW surface.^13^ With increasing operating temperature, surface traps become activated, leading to severe charge carrier loss. As we will show below, this is one of the main reasons that unpassivated (bare) thin NWs only have good optoelectronic performance at low temperature, which presents a challenge towards achieving high quantum efficiency at room temperature, and hinders the development of room-temperature high-performance optoelectronic and photonic devices. Due to these reasons, most studies of high-InAs-content NWs so far have been primarily focused on the fabrication of electronic devices,^14−16^ while the optoelectronic niche remains largely un-studied, especially for NWs with smaller diameters.

Surface passivation is thus critical for InAsP thin-NW devices to achieve high performance, but at this stage there is still a lack of an effective and stable passivation method. Chemical passivation, such as with sulfide solutions, can provide an effective instant passivation, which however lacks long-term stability.^17,18^ A more effective and robust passivation technique is using in-situ-grown inorganic materials with a wider band gap that can block carriers from reaching the surface states. However, to achieve effective passivation, a passivation layer with a thickness on the order of 10–100 nm is required to prevent carrier tunneling to the surface, which puts high requirements on lattice matching between the NWs and the passivation layers. Thus, the most widely used surface passivation methods for GaAs-based NWs utilize closely lattice-matched shells of a different material, including AlGaAs,^19−21^ AlInP,^22^ and InGaP.^23^ With the exception of GaAs/AlGaAs, achieving a lattice-matched shell composition requires careful tuning of growth parameters for each individual system. This is particularly challenging for NW growth owing to the different diffusion lengths of different adatoms on the substrate and nanowire surface, which can give rise to nonuniform composition both within and between different nanowires.^24^ For InAsP NWs, Al(Ga)AsSb is lattice matched but suffers a wide miscibility gap which makes growing the required alloy compositions extremely challenging. The use of a thick lattice-mismatched material will introduce large strain and seriously degrade the crystal quality.^25^ The strain induced by a thicker lattice-mismatched shell inevitably affects the band structure and NW morphology, limiting further device possibilities. Therefore, an alternative passivation method will be needed for InAsP NWs that requires no lattice-matching and has little impact on the other properties.

An alternative passivation route is to overcoat the NW with a material that itself has a low surface recombination velocity, so that recombination is reduced even when substantial tunneling into the layer occurs. Among the III–V materials, InP has an ultralow surface recombination velocity of ∼170 cm/s that is much lower than that of other III–Vs (e.g., GaAs ≈ 5.4 × 10^5^ cm/s).^10^ InP-based materials thus have been used widely for surface passivation.^26−28^ For example, Holm et al. covered their GaAsP NW solar cell with an additional shell of ∼10 nm InGaP and improved the efficiency from ∼6% to >10%.^29^ Considering the passivating nature of InP, it is highly likely that even an ultrathin layer of InP of a few nanometers can still have a substantial passivation effect, which may obviate the need for a thick passivation layer and hence circumvent the lattice-matching issue. This idea has been proven to be highly effective in improving the carrier mobility of InAs-based transistors by solving the surface-band bending issue, and reducing the surface roughness and detrimental effect of ionized impurity scattering centers.^30−32^ However, the performance of an electronic device relies only on the dynamics of one type of injected carrier (either electrons or holes); whereas optoelectronic performance is controlled by the complex interaction between both types of carriers. Optoelectronic performance therefore requires dedicated study beyond electronic studies alone. In addition, the long-term stability is critical for the lifetime of NW-based devices. So far, there is still a lack of detailed and systematic reports on whether the ultrathin InP layer can provide long-term protection to the optical and optoelectronic properties of passivated InAs-based NWs.

In this study, the long-term passivation effect of using ultrathin InP cladding layer is investigated for InAsP NWs of a small diameter (30–40 nm). Even after storing in an ambient atmosphere for over 3 years, this passivation technique still effectively reduces the surface recombination rate and enhances the carrier lifetime, leading to greatly improved optical properties with largely enhanced thermal stability.

The InAs_0.8_P_0.2_ NWs were grown by molecular beam epitaxy (MBE) without using foreign-metal catalysts. The majority of the NWs stand vertically on the silicon substrate with a very thin diameter of 30–40 nm and a length of ∼500 nm (Figure 1a). After cladding with a thin layer of InP, the NWs are slightly bent due to the introduction of strain (Figure 1b). The morphology and composition of the InAsP/InP was further analyzed using STEM along the ⟨112⟩ direction. Phosphorus maps and elemental distribution profiles in the radial direction of the NW reveal a thin P-rich shell 2–3 nm in thickness (Figure 1c and d), corroborated by the intensity profile taken in the NW radial direction in ⟨112⟩-Annular Dark Field images in Figure 1f. The InP shell was grown at an extremely low growth rate of 0.077 ML/s which is far lower than the normal growth rate of 1 ML/s. So, a uniform shell is expected. However, it may be affected by the shadowing effect of the neighboring NWs. So, the InP shell thickness was measured at the tip of NWs to identify the biggest thickness of the entire NW. Further optimization may be performed by using patterned substrates to allow accurate control of the interwire distance.^33^ The crystal structure was determined using images of NWs along ⟨110⟩ (Figure 1e). Higher magnification images of areas similar to the one enclosed in the red square confirmed the presence of stacking faults, with a mixture of wurtzite (WZ) and zincblende (ZB) phases, similar to previous reports.^34,35^ No dislocations were observed due to the small InP thickness (Figure 1g).

**Figure 1:**
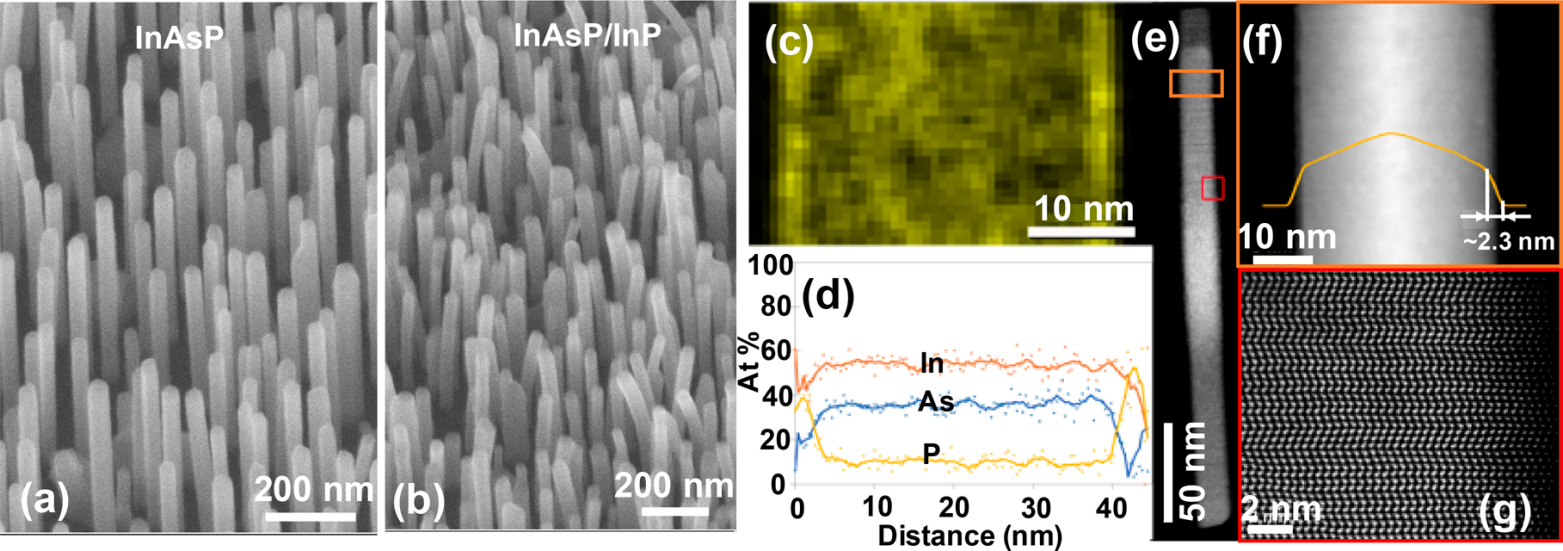
Morphology of InAsP NWs with and without InP passivation. (a) Bare InAsP NWs. (b) InAsP NWs cladded with ∼3 nm InP. (c–g) Measurement results from NWs in (b). (c) P map and (d) elemental distribution in a NW segment, confirming a P-rich shell. (e) ADF along ⟨110⟩ of a nanowire. (f) ADF-STEM image along the ⟨112⟩ direction revealing the NW shape with a shell thickness ∼2–3 nm. (g) Atomically resolved ⟨110⟩ ADF-STEM image of the NW area enclosed in the red square in part (e).

After being stored in an ambient atmosphere for over 3 years, optical pump–terahertz probe spectroscopy (OPTP) was performed to probe bare and passivated InAsP nanowires to study the influence of the ultrathin InP surface passivation layer on the dynamics of the photogenerated carriers. The bare and passivated nanowires were placed on individual quartz substrates and were photoexcited with an optical pump pulse centered at 2060 nm (0.6 eV) just above the bandgap of the InAsP core. This excitation energy selectively photoexcites the core but not the InP shell. This allows effective comparison of the charge carrier dynamics of the passivated and unpassivated nanowires. The photoexcitation pulse induces a change in the transmission of the terahertz (THz) probe pulse, Δ*E*. The measured Δ*E* is directly proportional to the photoconductivity change (Δσ) of the nanowires.

The room-temperature photoconductivity (Δσ) decays, obtained from the OPTP measurements on InAsP and InAsP/InP nanowires, are presented in Figure 2a. The Δσ decays shows that the passivated nanowires have slower recombination in contrast to the rapid Δσ decay of the bare InAsP nanowires. This comparison suggests that surface traps are responsible for the rapid Δσ decay of bare InAsP nanowires and that these traps are effectively reduced by passivating with a thin InP layer. The Δσ decay of the bare InAsP nanowires is well-fitted by a monoexponential function, which is further evidence that charge carriers recombine rapidly through trap-assisted Shockley–Read–Hall mechanisms. The Δσ decay of the passivated InAsP/InP nanowires deviates from a monoexponential function and slows with time after photoexcitation, indicating that the remaining traps may saturate as they are filled. The photoconductivity decays were fitted at early times after photoexcitation to assess the surface recombination rate when the surface traps are originally unoccupied. The decays were fitted with monoexponential functions yielding charge carrier lifetimes τ of ∼120 ps for the bare InAsP nanowires and ∼340 ps for the passivated nanowires, corresponding to an increase in the carrier lifetime by a factor of ∼3. This highlights the effectiveness of the ultrathin passivation layer in reducing surface state density in the InAsP nanowires. The effective recombination time in nanowires in closely approximated by
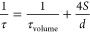
1where *d* is the nanowire diameter, *S* is the surface recombination velocity, and *τ*_volume_ is the time constant for recombination within the nanowire’s volume. By fitting eq 1 to the experimental charge carrier lifetime τ values, and assuming *τ*_volume_ is the same for passivated and unpassivated nanowires, we calculate that the InP surface passivation lowers the surface recombination velocity in the InAsP nanowires by Δ*S* = 6.1 × 10^3^ cm/s compared to the bare InAsP nanowires, thereby allowing an increase in the fraction of radiative recombination in the InAsP nanowires. To estimate the surface recombination velocity in the passivated nanowires, *S*_InAsP/InP_ = *S*_InAsP_ – Δ*S,* we consider Δ*S* and the surface recombination velocity in unpassivated nanowires, *S*_InAsP_. As the composition of the unpassivated InAsP nanowires is close to that of InAs nanowires, it is expected that the surface recombination velocity *S* value should be close to or less than that of InAs nanowires, which we measure to be *S*_InAs_= 8.7 × 10^3^ cm/s (Supporting Information Figure S1) consistent with previous reports.^10^ Assuming *S*_InAsP_ ≲ *S*_InAs_= 8.7 × 10^3^ cm/s and Δ*S* of 6.1 × 10^3^ cm/s, an approximate upper limit of *S*_InAsP/InP_ = 2.6 × 10^3^ cm/s is extracted for the passivated InAsP/InP nanowires. The increase in charge carrier lifetime and decreased surface recombination velocity indicates a large reduction in the surface state density by the highly effective ultrathin InP shell.

**Figure 2:**
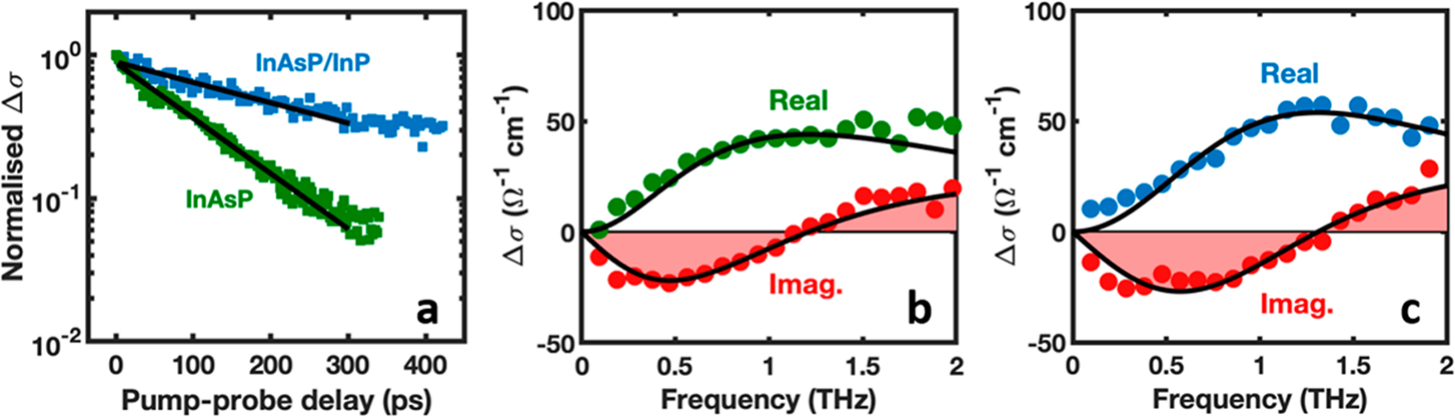
Influence of the InP surface passivation layer on carrier dynamics. (a) Normalized photoconductivity decays of InAsP and InAsP/InP nanowires on a semilogarithmic scale. The lines are monoexponential fits to the decay. Photoconductivity spectra of (b) InAsP and (c) InAsP/InP nanowires at a time delay of 10 ps after photoexcitation. Dots represent data points and black solid lines are Lorentzian fits.

The photoconductivity spectra of both nanowire samples shown in Figure 2b and c were measured at 10 ps after photoexcitation, and each spectrum was fitted with a Lorentzian function:
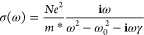
2This Lorentzian response is typical of plasmon modes of semiconductor nanostructures. For the effective mass *m*_e_^*^, we use the value for bulk InAsP, namely, *m*_e_^*^ = 0.0347*m*_e_.^36^ The measured photoconductivity Δσ is predominantly from electrons, since the effective hole mass in InAs is significantly larger than that of the electrons. From the fitting, the carrier scattering rates and electron mobilities are extracted. The extracted carrier mobilities are ∼3200 cm^2^ V^–1^ s^–1^ for both the passivated nanowires and the bare InAsP nanowires, thus showing that there is no significant degradation in mobility caused by lattice mismatch-induced strain between the core and the shell. Previous field-effect transistor (FET) measurements on InAs nanowires have reported increases in transconductance with InP passivation, attributed to the reduction of the electron accumulation layer at the surface (see ref (32)). It should be noted that unlike FET measurements, our OPTP measurements of mobility do not require approximations of the gate oxide capacitance and are therefore free of any systematic errors in mobility that can be introduced by this term. In the present study, regardless of passivation, the measured mobilities are an order of magnitude lower than those of bulk InAs (40,000 cm^2^ V^–1^ s^–1^) which may be due to the presence of stacking faults in these NW structures.^37^ Compositional fluctuations in the axial direction are also possible, which may cause additional carrier scattering.^38^

The power-dependent performance of the InAsP NWs with and without surface passivation was studied by photoluminescence (PL) measurements. The power-dependent PL spectra obtained at 12 K are shown in Figure 3a and b. The unpassivated NWs exhibit a broad PL spectrum peaking between 2400 and 2600 nm (0.48 to 0.52 eV). In contrast, the InP-passivated NWs (Figure 1b) exhibit a blue-shifted peak centered at 2200 nm (0.56 eV) due to strain caused by the InP passivation, which is in accordance with the slight NW bending.^39,40^ As can be seen in Figure 3a and c, the PL intensity of the bare NWs increases with excitation power at low excitation power range, but saturates and then decreases rapidly above 600 mW. This saturation is attributed to laser excitation-induced wire heating which activates surface traps to increase nonradiative recombination. At low excitation powers, the wire laser-heating effect is small, the diffusion of the carriers is slow, and hence a larger fraction of carriers recombine radiatively inside the NWs, which allows the emission intensity to increase with the excitation power. At high excitation power, the wire is heated to higher temperatures, and enhanced diffusion drives carriers to the surface, where they are captured by the high-density surface traps to reduce radiative recombination efficiency. Thus, the nonradiative carrier loss increases severely, and hence the emission intensity reduces with the increase of the excitation power. After the surface passivation, the carrier loss is greatly reduced, and the NWs do not show the phenomenon of reduced PL intensity at high excitation powers, which can be seen in Figure 3b and c.

**Figure 3:**
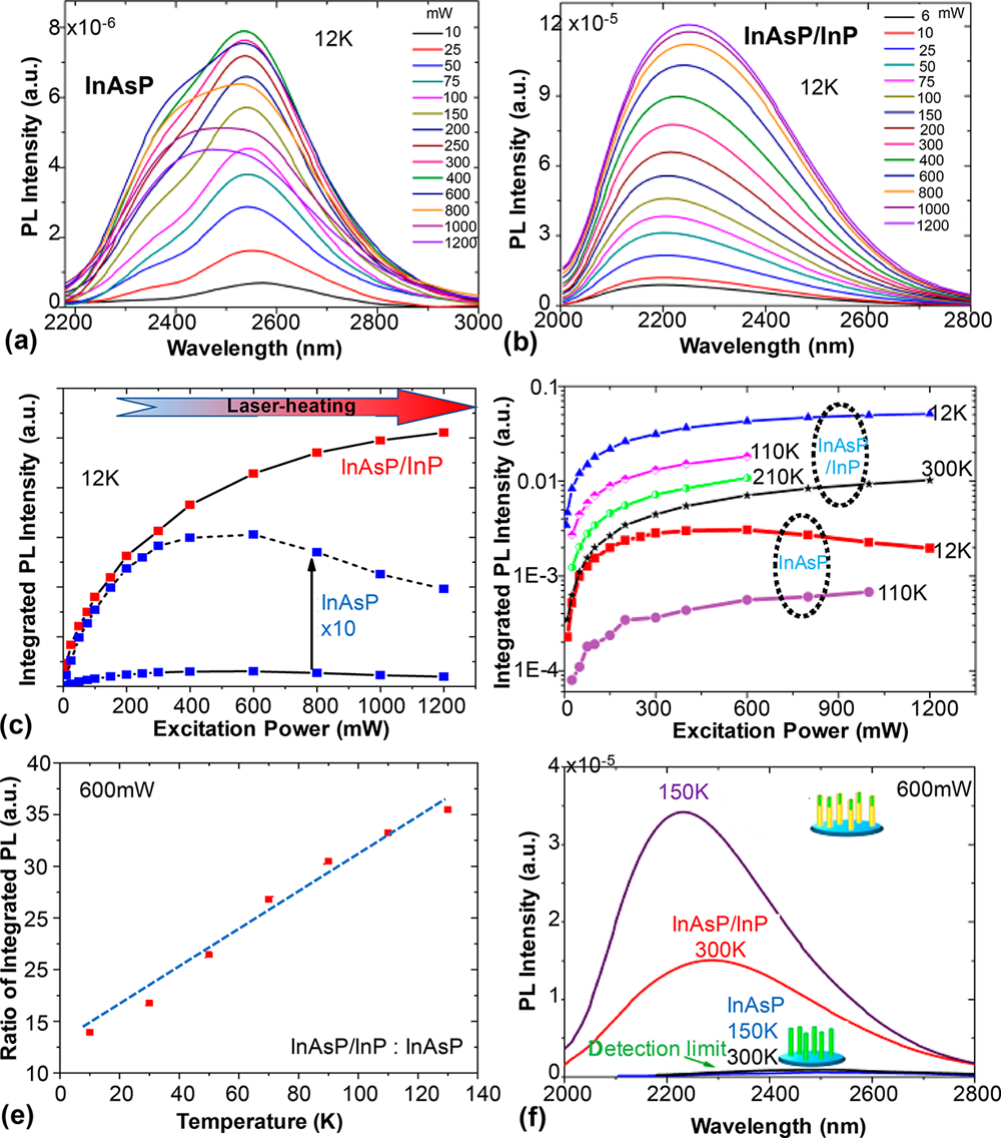
Thermal tolerance of InAsP NWs with and without surface passivation. (a) and (b) are the power-dependent PL spectra of InAsP and InAsP/InP NWs at 12 K, respectively. (c) Integrated PL intensity as a function of excitation power at (c) 12 K and (d) different temperatures. (e) Integrated PL intensity ratio between passivated and bare NWs (InAsP/InP/InAsP) as a function of temperature at 600 mW excitation power. (f) PL spectra of both samples measured at 150 and 300 K.

The power-dependent performance of the bare NWs changes significantly with temperature. As can be seen in the low excitation power region in Figure 3d, the integrated emission intensity increases rapidly with the excitation power at 12 K, while the increase is much slower at 110 K and the intensity is 1 order of magnitude lower. This suggests that the increased temperature, and hence thermal energy, is enough to cause severe charge carrier loss at the surface. In contrast, the passivated NWs show almost identical power-dependent performance from low temperature to room temperature, and their emission intensity at room temperature is stronger than that of the bare NWs at 12 K. With the increase in temperature, the emission intensity ratio between the bare and passivated NWs increases almost linearly, which can be seen in Figure 3e. The passivated NWs still show strong emission at room temperature, while the emission of bare NWs is below our system detection limit for temperatures above 150 K (Figure 3f). All these observations further suggest that the surface passivation by thin InP can greatly improve the optoelectronic performance of the NWs at high temperatures and high carrier densities, which is highly important for practical applications of narrow-band gap semiconductor NWs.

In conclusion, thin InAsP NWs (30–40 nm diameter) without surface passivation are highly sensitive to the thermal environment, and their performance degrades rapidly with temperature, making their PL emission quench at ∼150 K and preventing room temperature applications. An ultrathin InP layer (2–3 nm) with good surface properties, e.g., low surface state density, can be used to passivate the NW surface, and its effectiveness and long-term stability have been investigated systematically after these samples have been stored in atmosphere for over 3 years. The InP passivation layer does not have any observable defects, despite the large lattice mismatch with the core NWs, due to being very thin. Upon passivation, the density of surface states dramatically decreases by at least 70%, and the lifetime and mobility of the carriers increases by a factor of ∼3. These improvements lead to greatly enhanced temperature/thermal stability and significantly expands the maximum operating temperature from ∼130 K to room emperature. With this efficient passivation technique, the performance of the NWs at room temperature is superior to that of the bare NWs at 12 K. This study provides an effective and long-term passivation method for narrow-band gap semiconductor NWs to realize high-temperature performance.

## Methods

### NW Growth

The InAsP NWs were grown directly on Si substrates by solid-source III–V molecular beam epitaxy without using foreign catalytic metals. The core InAsP NWs were grown with an In beam equivalent pressure, V/III flux ratio, P/(As+P) flux ratio, and substrate temperature of 2.75 × 10^–8^ Torr, ∼200, 60%, and ∼455 °C, respectively. The passivation InP shells were then grown with an In beam equivalent pressure, V/III flux ratio, and substrate temperature of 2.75 × 10^–8^ Torr, and ∼90 and ∼400 °C, respectively. The substrate temperature was measured by a pyrometer. The low temperature for shell growth is for achieving uniform shells. The NW sidewall is normally {110} which has very low surface energy. Adatoms are much more mobile on this surface than on the commonly used {100} facets. Therefore, a low temperature is necessary to reduce the mobility of adatoms and enhance their nucleation ability.^41^

### Scanning Electron Microscope (SEM)

The NW morphology was measured with a Zeiss XB 1540 FIB/SEM system.

### Transmission Electron Microscopy (TEM)

Simple scraping of the NWs onto a lacey carbon support was used to prepare TEM specimens. The TEM measurements were performed with a JEOL 2100 and doubly-corrected ARM200F microscopes, both operating at 200 kV.

### Optical Pump–Terahertz Probe Spectroscopy (OPTP)

An amplified Ti:sapphire laser with 8 W average power was used to generate 35 fs pulses centered at 800 nm. The optical alignment follows that described in ref (42) for OPTP spectroscopy. THz pulses were generated by optical rectification in a ZnTe (110) crystal. The nanowire sample was photoexcited by an optical pump pulse centered at 2060 nm (0.6 eV). The electric field of the THz pulse transmitted through the sample was detected by electro-optic sampling using a ZnTe (110) crystal and a balanced photodiode circuit.

### Photoluminescence (PL)

A mid-IR PL setup has been used to analyze the photoluminescence properties of both samples. A 532 nm laser was used to excite the samples and a liquid nitrogen cooled InSb detector to record the photoluminescence.

## Supporting Information Available

Photoconductivity decay data for unpassivated InAs nanowires (PDF)
