# Supplementary material for: Long-Term Stability and Optoelectronic Performance Enhancement of InAsP Nanowires with an Ultrathin InP Passivation Layer
Source: Nano Lett. 2022 Apr 14;22(8):3433–9. doi: 10.1021/acs.nanolett.2c00805 (PMC9097579; doi:10.1021/acs.nanolett.2c00805)
Supplement: Supplementary file 1 — nl2c00805_si_001.pdf [file nl2c00805_si_001.pdf]

Long-Term Stability and Optoelectronic Performance Enhancement of InAsP Nanowires with an Ultra-Thin InP Passivation Layer

LuLu Chen,[†⊥] Stephanie O. Adeyemo,[‡⊥] H. Aruni Fonseka,[#] Huiyun Liu,[§] Srabani Kar,[‡] Hui Yang,[‖*] Anton Velichko,[¶] David J. Mowbray,[¶] Zhiyuan Cheng,[†] Ana M. Sanchez,[#] Hannah J Joyce,[‡*] Yunyan Zhang[†§*]

† School of Micro-Nano Electronics, Zhejiang University, Hangzhou, Zhejiang, 311200, China

‡ Electrical Engineering Division, Department of Engineering, University of Cambridge, 9 JJ Thomson Avenue, Cambridge CB3 0FA, United Kingdom;

# Department of Physics, University of Warwick, Coventry CV4 7AL, United Kingdom;

§ Department of Electronic and Electrical Engineering, University College London, London WC1E 7JE, United Kingdom;

‖ Department of Materials, Imperial College London, Exhibition Road, London SW7 2AZ, United Kingdom

¶ Department of Physics and Astronomy and the Photon Science Institute, University of Sheffield, Sheffield S3 7RH, U.K.

⊥ These authors contributed equally to the work.

Supporting Information:

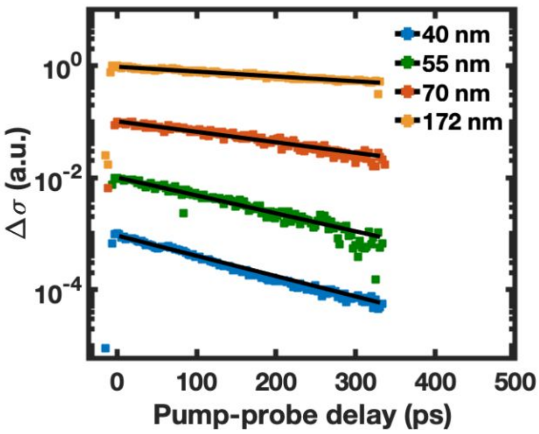

Figure S1. Photoconductivity decays of InAs nanowires of different diameters of 40, 55, 70 and 172 nm on a semilogarithmic scale fitted with lifetimes of τ = 120, 135, 230 and 505 ps respectively. The black lines are monoexponential fits to the decay.

Figure S1 plots photoconductivity decay curves obtained from InAs nanowires of different diameters. These data were obtained by optical pump–terahertz probe spectroscopy as described in the main manuscript. Charge carrier lifetimes τ were extracted by fitting monoexponential decays to the data. By fitting Equation (1) (main manuscript) to the charge carrier lifetimes τ as a function of diameter *d*, a surface recombination velocity S_{InAs} value of 8.7×10^3 cm/s is obtained which is the same order of magnitude as that in previous reports.[1]

References:

(1) Joyce, H. J.; Docherty, C. J.; Gao, Q.; Tan, H. H.; Jagadish, C.; Lloyd-Hughes, J.; Herz, L. M.; Johnston, M. B. Electronic Properties of GaAs, InAs and InP Nanowires Studied by Terahertz Spectroscopy. *Nanotechnology* **2013**, *24* (21), 214006. https://doi.org/10.1088/0957-4484/24/21/214006.
